# Antimicrobial susceptibility patterns: a three-year surveillance study in a rehabilitation setting

**DOI:** 10.11604/pamj.2016.23.214.8474

**Published:** 2016-04-22

**Authors:** Liaqat Ali Chaudhry, Jaffar Ali Al-Tawfiq, Marwan Mohammed Zamzami, Saeed Abdullah Al-Ghamdi, Asirvatham Alwin Robert

**Affiliations:** 1 Department of Internal Medicine, Pulmonary Division, Sultan Bin Abdulaziz Humanitarian City, Riyadh, Kingdom Saudi Arabia; 2 Speciality Internal Medicine, John Hopkins Aramco Healthcare, Dhahran, Kingdom Saudi Arabia; 3 Department of Orthopedic Surgery, College of Medicine, King Saud University, Riyadh, Kingdom Saudi Arabia; 4 Department of Laboratory Services, Sultan Bin Abdulaziz Humanitarian City, Riyadh, Kingdom Saudi Arabia; 5 Department of Endocrinology and Diabetes, Diabetes Treatment Center, Prince Sultan Military Medical City, Riyadh, Kingdom Saudi Arabia

**Keywords:** Antimicrobial susceptibility, resistance patterns, multi-drug resistant organisms

## Abstract

**Introduction:**

To analyze the susceptibility patterns in a rehabilitation center.

**Methods:**

This retrospective observational study was conducted between January 2011 and to January 2013 at Sultan Bin Abdulaziz Humanitarian City (SBAHC), Riyadh, Kingdom of Saudi Arabia. Number of the patients, specimen type, pathogen detected and antibiogram were entered in database for analysis using Inter System Track care software.

**Results:**

A total of 4525 isolates were available from 5148 patients. Most (74%) of the isolates were from urine samples and were due to *Eschericia coli* (49.8%), *Enterococcus faecalis* (15%) and (*Proteous mirabilis*(9.49%). Of all the isolates, *Eschericia coli* was the commonest (49.8%) Gram negative organism, while*(Stahylococcus aureus was the commonest* (51%) among Gram positive organisms. The most effective antibiotics against *Pseudomonas aeroginosa* were ciprofloxacin and gentamicin. Meropenem shows excellent activity against Gram negative bacteria. Methicillin resistant *Staphylococcus aureus* (MRSA) was susceptible to Vancomycin and Rifampicin in 97% and 85% cases.

**Conclusion:**

A high incidence of urinary tract infections caused by *Eschericia coli, Enterococcus faecalis* and *Proteous mirabilis* was reported. *Staphylococcus aureus* was the commonest pathogen isolated from infected bed sores.

## Introduction

The pre-antibiotic era was characterized by high morbidity and mortality rates; however, over the last several decades, the rise in antimicrobial use and guidelines for non-concordant therapies has resulted in a dramatic increase in the development of many resistant pathogen strains. Thus there is an increase in treatment failure rates, mortality and cost of management [1–3]. Apart from the naturally occurring biological mutations other contributing factors include, poor case management by unsuitable combinations and subtherapeutic doses of antimicrobial agents. In fact, it is the DNA (deoxy nucleic acid) mutations [4] which control the resistance to antimicrobials, at the time of the plasmid transfer between the bacterial cells through conjugation [5]; however, the enzymatic de-activation of the antibiotics [6, 7], a change in their action sites, both quantitatively and qualitatively [8], the formation of the extended spectrum beta-lactamase enzyme (ESBL) and antibiotic efflux from the cell are a few of the other significant mechanisms involved [9]. E. coli and Klebsiella pneumoniae are some strains which exhibit cross reactivity against a wide range of antimicrobials [10, 11]. The objective of this study was to analyze the antibiograms during three years period at Sultan Bin Abdulaziz Humanitarian City (SBAHC), Riyadh, Kingdom of Saudi Arabia.

## Methods

This retrospective observational study was conducted to analyze the susceptibility patterns of all the isolates extended over a three years period from January 2011 to January 2013. Each isolate was tested for susceptibility against a specific antimicrobial agent with a single disc((manufacturer OXOID LTD). Interpretation of susceptibility results were done as recommended by the clinical and laboratory standard institute (CLSI). Organisms used as control include *Staphylococcus aureus* (ATCC25923), *Eschericiacoli* (ATCC25922), *Pseudomonas aeruginosa* (ATCC27853), *Enterococcus faecalis* (ATCC9212) and Klebsiella pneumonia (ATCC13883). Along with the susceptibility tests performed on the clinical isolates, the zone diameters were also recorded in line with the CLSI norms. Vancomycin susceptibility results are re-checked by automated, as well as manual methods and are verified by the regional reference laboratory. An inhibitory concentration of vancomycin used for *Staphylococcus aureus* is 6 micrograms/ml by agar dilution method in BHI agar. An inoculum from a 0.5micro Farland turbidity suspension using a micropipte is done, with 10 microlitre drop on spot area of 10-15mm of the plate. Readings are done, with transmitted light after an incubation period of 24 hours at a temperature of 35c. The total number of patients, specimen type, pathogen detected and antibiograms were entered in a data base and were analyzed with the inter system track care software.

## Results

**Figure 1:**
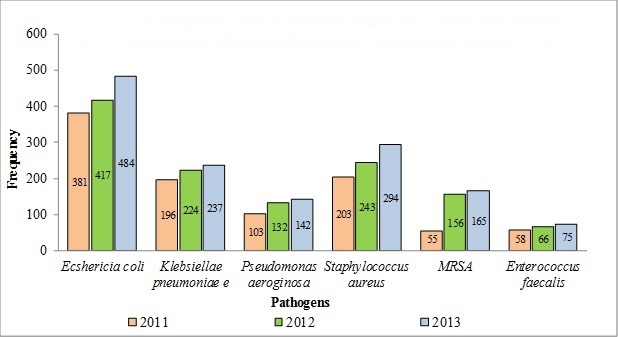
Type and frequency of pathogens isolated during 2011-2013

During the three-year period, 4525 susceptibility tests were performed on specimens taken from 5148 patients. The eight most common pathogens isolated are shown in Table 1. *Staphylococcus aureus* was the most common (51%) organism among 1449 Gram positive organisms, mainly from infected bed sores. On the other hand among 2570 Gram negative isolates the most frequent pathogen *were Eschericia coli* (49.8%). Other pathogens isolated from urine being Gram positive *Enterococcus faecalis* (15%) and *Proteous mirabillis*(9.5%). Susceptibility patterns of various antimicrobial agents are shown in Table 2 and Table 3. The most effective antibiotics against *Pseudomonas aeroginosa* were ciprofloxacin and gentamicin. Meropenem shows excellent activity against Gram negative bacteria. Methicillin resistant *Staphylococcus aureus* (MRSA, n = 376) was susceptible to Vancomycin and Rifampicin in 97% and 85% cases. The type and frequency of pathogens isolated during 2011-2013 are shown in Figure 1.

**Table 1:** The eight most common pathogens isolated during 3 year period between January 2011 and January 2013

Organisms	N (%)	Organisms
*EschericiaColi*	1282 (49.8%)	*Providencia Stuartii 80(7.53%)*
*Klebsiella pneumoniae*	657 (25.5%)	*Mrgagnella morgagnii 37(3.1%)*
*Pseudomonas aeroginosa*	387 (15%)	*Enterobacter aerogenes 58(5.71%)*
*Proteous mirabillis*	244 (9.49%)	*-*
*Staphylococcus aureus*	740 (51%)	*-*
*Methicillinresistance Staphylococcus aureus*	376 (26%)	-
*Enterococcus faecalis*	220 (15.1%)	-
*Coagulase negative Staphyslococcus aureu*	113 (7.8%)	-
*Enterococcus faecium maltophia*	5 (1.14%)	-
*Streptococcus pyogenes*	8 (1.39%)	-
*Streptococcus groupc/g*	5 (0.69%)	-
*Streptococcus uberis*	1 (0.19%)	-
*Streptococcus species*	4 (1.0%)	-
*Streptococcus alagactiae*	26 (4.89%)	-
*Streptococcus pneeumoniae*	8 (1.79%)	-

**Table 2:** Susceptibility patterns of common Gram negative bacteria

	Ampicillin	Trimethoprim-sulfamethoxazole	Meropenem	Piperacillin-tazobactam	Ciprofloxacin	Gentamicin	Nitrofurantoin
*Eschericia coli*	1282 (23%)	1282 (44%)	1282 (99%)	1282 (86%)	1282 (39%)	377 (86%)	1282 (81%)
*Klebsiella pneumoniae*	-	657 (56%)	657 (98%)	657 (69%)	657 (59%)	627 (66%)	657 (37%)
*Proteous Mirabillis*	-	341 (23%)	341 (100%)	341 (79%)	347 (32%)	341 (44%)	341 (3%)
*Pseudomonas aeroginosa*	-	377 (6%)	377 (87%)	377 (53%)	377 (86%)	277 (95%)	-

**Table 3:** Susceptibility Patterns of common Gram Positive bacteria

	Ampicillin	ciprofloxacin	Oxacillin	Clindamycin	Vancomycin	Rifampicin	Cefazolin	Nitrofurantoin
*Enterococcus faecalis*	202 (98%)	-	-	-	-	-	-	220 (84%)
*Staphylococcus aureus*	-	740 (9%)	740 (100%)	740 (95%)	740 (98%)	740 (1%)	-	
*Coagulase negative Staphylococcus*	-	-	113 (67%)	113 (87%)	113 (97%)	-	113 (80%)	
*MRSA*	-	-	-		376 (97%)	376 (85%)	-	

## Discussion

Among a total of 1449 Gram-positive organisms mainly from bedsores, *Staphylococcus aureus* ranked the commonest organism and *Eschericia coli* were the most frequent isolated Gram-negative organisms mainly from the urine. *Streptococcus pneumonia* was reported in only 1.2% of tests compared with the reports from an acute care hospital where it was the commonest pathogen (23.4%) [12]. Ciprofloxacin susceptibility was 86% for *Pseudomonas aeroginosa* and 59% for *Klebsiella pneumonia*. However a low susceptibility to ciprofloxacin was observed for Staphylococcus aureus (9%), followed by Methicillin resistant *Staphylococcus aureus* (28%). Only 32% of Proteuos mirabillis and 39% of Echericia coli were susceptible to ciprofloxacin. Gentamicin susceptibility was 95% for Pseudomonas aeroginosa and 56% for Eschericia coli. Nitrofurantoin susceptibility was 84% for *Enterococcus faecalis* and 81% for *Eschericia coli. Enterococcus faecalis* was sensitive to ampicillin in 98% cases, while ampicillin was affective only in 1% for *Klebsiellae pneumoniae*, 23% for Eschericia coli and 29% for *Proteous mirabillis*. A low susceptibility of *Klebsiellae pneumoniae* to ampicillin has also been reported from an acute care hospital setting [12]. *Enterococcus faecalis* was sensitive to ampicillin in 98% cases, while most of other organisms causing urinary tract infections were found less susceptible to ampicillin like *Klebsiella pneumniae* (11%), Ecoli (23%) and *Proteous mirabillis* (29%). Till date, meropenem and tazocin ranked as the most affective antimicrobial agents against a multitude of pathogens including *Pseudomonas aeroginosa*. Clindamycin remains an affective choice against *Staphylococcus aureus (95%) as well as Coagulase negative Staphylococcus aureus* (87%) isolated mainly from bed sores, in 100% and 67% cases respectively. Among susceptible organisms to Vancomycin include *Staphylococcus aureus* (100%), MRSA (97%) and Coagulase negative *Staphylococcus aureus* (96%). While, organisms susceptible to Rifampicin include *Staphylococcus Aureus* (100%), MRSA (85%) and *Coagulase negative Staphylococcus* (47%). Another antimicrobial agent Cefazolin, still being used as first choice pre-surgical prophylaxis retains its merit, based on its efficacy against skin related pathogens, like *Staphyloccoccus coagulase negative* (80%) Table 2. Methicillin resistant *Staphylococcus aureus* (MRSA, n = 376) was susceptible to Vancomycin and Rifampicin in 97% and 85% cases Table 3. Another significant occurrence was, a fairly high increase in all pathogens types in general, while a specific rise was noted in the frequency of the organisms causing urinary tract infections like *Eschericia coli, Klebsiella pneumoniae, Pseudomonas aeruginosa, Staphylococcus aureus, MRSA andEnterococcus faecalis* especially towards the end of year 2012 and onwards Figure 1. This increase indicates higher incidence of urinary tract infections among our patients, having chronic neurological conditions like neurogenic urinary bladder associated with conditions like stroke, traumatic brain injury, children with cerebral palsy, and spinal cord injuries requiring either indwelling foley's catheters or being on 4 hourly clean intermittent catheterization program (CIC).

Yet another significant contributing factor could be attributed possibly to the recent expansion in bed capacity of our hospital from 312 beds to 450 beds in late year 2012 including chronic wound care unit having 10 beds. A significant number of admitted patients in our hospital have recent history of hospitalization in addition to direct hospital to hospital transfer of patients. True to the well-known saying ‘prevention is better than cure’ there is an urgent need to implement measures to stop antibiotic resistance from being built. Issues such as antimicrobial resistance become especially significant to hospitalized patients, because these are the persons who are usually compromised by different co-morbid factors like advanced age, serious illnesses, immunosuppressive therapy involving invasive procedures, catheterizations, admission to HDU (High Dependency Unit) and necessitating mechanical ventilator support etc. Such patients are highly vulnerable and pick up more infections and develop drug resistant micro-organisms readily [13]. It is easier to manage hospitals than open communities, by monitoring antibiotic usage as well as incorporating strong preventive and infection control measures to limit the spread of infections. Several measures have been suggested to reduce the colonization or infections due to drug resistant pathogens in hospitalized patients. They are, administering antibiotics which have no resistant pathogens, reducing the usage of drugs having known high resistance, improving the methods of infection control, implementing hand hygiene culture among the health care workers, isolating, co-horting strategies, and finally, increasing patient turnover and reducing the rate of admission in hospitals or HDU of those patients carrying resistant bacteria [14]. The commonest pathogens causing urinary tract infections and infected bed sores are, *Eschericia coli* (49.8%) and *Staphylococcus aureus* (16.8%). Almost identical results have been reported in other rehabilitation facilities [15]. However, in our study urinary tract infections has shown an upward trend (Figure 1). Guidelines non-concordant uses of antimicrobial agents, unrealistic antibiotic prescriptions and irresponsible attitudes, have been attributed to both physicians and patients. Physicians either lack the time to explain the indications or contraindications of the antibiotics to their patients, or they lack knowledge and judgment as to when to prescribe them and when not. At times, it is the patient who insist on taking the antibiotics [16].

## Conclusion

The antibiograms studied in our hospital during three years of study, reveal a high incidence of urinary tract infections, often accompanied by hospital associated pathogens, which were difficult to treat with the routine necessary antimicrobial agents. This was on one hand due to the increase in the number of patients having a recent history of hospitalization; and on the other hand due to chronic neurological conditions requiring urinary catheterization and therefore urinary tract infections. Secondly many patients are bed ridden having hospital acquired pressure ulcers (HAPU),which get infected. A HAPU prevention program could again help to minimize development of bed sores and related infections. In the light of these observations, we in our facility has adopted an antibiotic stewardship program limiting the indiscriminate use of meropenem, tazocin and aminoglycosides like antibiotics at the junior level, unless recommended by the relevant consultants. At the same time a strong and continuous HAPU prevention campaign has been launched in our hospital

### What is known about this topic

- Antibiotic resistance is increasing worldwide;
- Antibiotic resistance pattern is well known for acute setting;
- Emerging multi-drug resistant organisms constitute a threat to the public.

### What this study adds

- Evaluation of antibiogram in a rehabilitation center;
- Demonstration of increased resistance in rehabilitation center;
- Similar observation of increasing Escherichia coli and Staphylococcus aureus.
